# Supplementary material for: Prognostic Factors of Long-Term Outcomes after Primary Chemo-Radiotherapy in Non-Metastatic Anal Squamous Cell Carcinoma: An International Bicentric Cohort
Source: Biomedicines. 2023 Mar 6;11(3):791. doi: 10.3390/biomedicines11030791 (PMC10045746; doi:10.3390/biomedicines11030791)
Supplement: Supplementary file 1 [file biomedicines-11-00791-s001.zip › biomedicines-2213142-supplementary.pdf]

Supplementary Material

Prognostic Factors of Long-Term Outcomes After Primary Chemo-Radiotherapy in Non-Metastatic Anal Squamous Cell Carcinoma: An International Multicenter Cohort.

Supplementary Table S1: Univariate analysis Complete Response based on patient demographic and clinical characteristics

**Patient characteristics**	**All**	**Complete Response**	**Non-complete Response**	**x2**	***p-value***
	n=176 (%)	n=123 (%)	n=53 (%)		
Gender					
Male	57 (32)	36 (29)	21 (40)	1.81	0.17
Female	119 (68)	87 (71)	32 (60)		
Tumor					
T1-T2	76 (43)	59 (48)	17 (32)	3.81	**0.05**
T3-T4	100 (57)	64 (52)	36 (67)		
Nodes					
Negative	81 (46)	64 (52)	17 (32)	5.93	**0.01**
Positive	95 (54)	59 (48)	36 (68)		
HIV					
Negative	138 (78)	100 (81)	38 (72)	2.02	0.15
Positive	38 (22)	23 (19)	15 (28)		
HPV					
Negative	12 (7)	7 (6)	5 (9)	0.82	0.37
Positive	164 (93)	116 (94)	48 (91)		
HIV-HPV infection					
HIV- and HPV-	10 (6)	5 (4)	5 (10)	*3.42	*0.06
*HIV- and HPV+	128 (73)	95 (77)	33 (62)		
HIV+ and HPV-	2 (1)	2 (2)	0		
*HIV+ and HPV+	36 (20)	21 (17)	15 (28)		
Differentiation					
Well	29 (16)	22 (18)	7 (15)	1.45	0.48
Moderate	86 (49)	60 (49)	26 (54)		
Poorly	40 (23)	25 (20)	15 (31)		
Unknown	21 (12)	16 (13)	5 (31)		

x2: Pearson's Chi-squared test

* Statistically significant differences among treatments (p<0.05).

Supplementary Table S2: Univariate analysis Overall Survival based on patient demographics, clinical and treatment characteristics

**Patient characteristics**	**All**<br>n=175 (%)	**No death**<br>n=143 (%)	**Death**<br>n=32 (%)	**x2**	***p-value***
Gender					
Male	57 (33)	42 (29)	15 (47)	3.64	**0.05**
Female	118 (67)	101 (71)	17 (53)		
Tumor					
T1-T2	76 (43)	68 (48)	8 (25)	5.41	**0.02**
T3-T4	99 (57)	75 (52)	24 (75)		
Nodes					
Negative	80 (46)	69 (48)	11 (34)	2.02	0.15
Positive	95 (54)	74 (52)	21 (66)		
HIV					
Negative	137 (78)	114 (80)	23 (72)	0.94	0.33
Positive	38 (22)	29 (20)	9 (28)		
HPV					
Negative	11 (6)	9 (6)	2 (6)	0.00	0.99
Positive	164 (94)	134 (94)	30 (94)		
HIV-HPV infection					
HIV- and HPV-	9 (5)	7 (5)	2 (6)	*1.38	*0.24
*HIV- and HPV+	128 (73)	107 (75)	21 (66)		
HIV+ and HPV-	2 (1)	2 (1)	0		
*HIV+ and HPV+	36 (21)	27 (19)	9 (28)		
Differentiation					
Well	28 (16)	25 (17)	3 (10)	11.3	**0.003**
Moderate	86 (49)	74 (53)	12 (37)		
Poorly	40 (23)	25 (17)	15 (47)		
Unknown	21 (12)	19 (13)	2 (6)		

x2: Pearson's Chi-squared test

* Statistically significant differences among treatments (p<0.05).

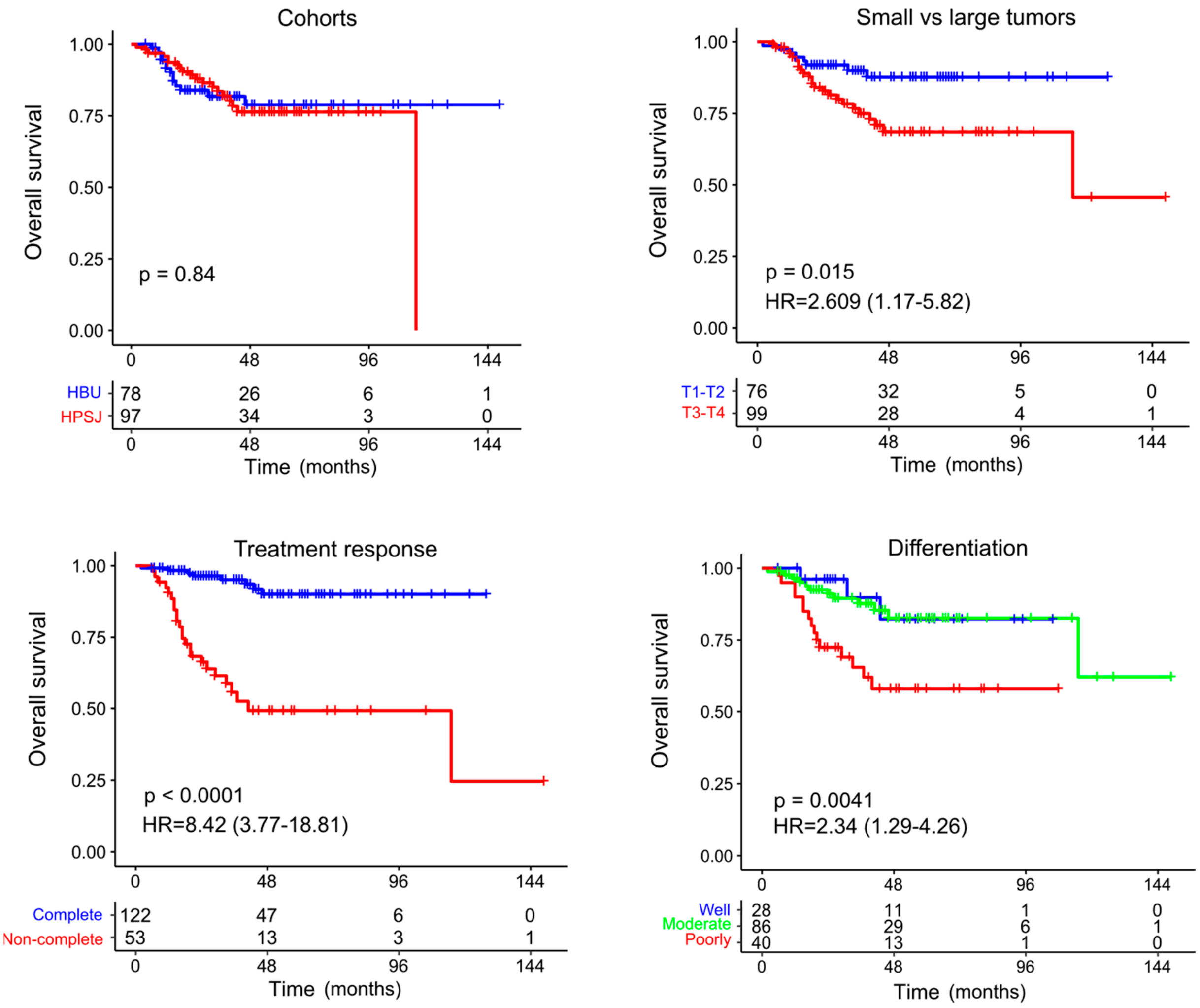

Supplementary Figure S1. Overall Survival Kaplan–Meier survival analysis and Cox regression analysis of NM-ASCC patients according to clinicopathological features.
